# Supplementary material for: Amino Acid Properties, Substitution Rates, and the Nearly Neutral Theory
Source: Genome Biol Evol. 2025 Feb 20;17(3):evaf025. doi: 10.1093/gbe/evaf025 (PMC11884779; doi:10.1093/gbe/evaf025)
Supplement: evaf025_Supplementary_Data [file evaf025_supplementary_data.zip › Supplement.docx]

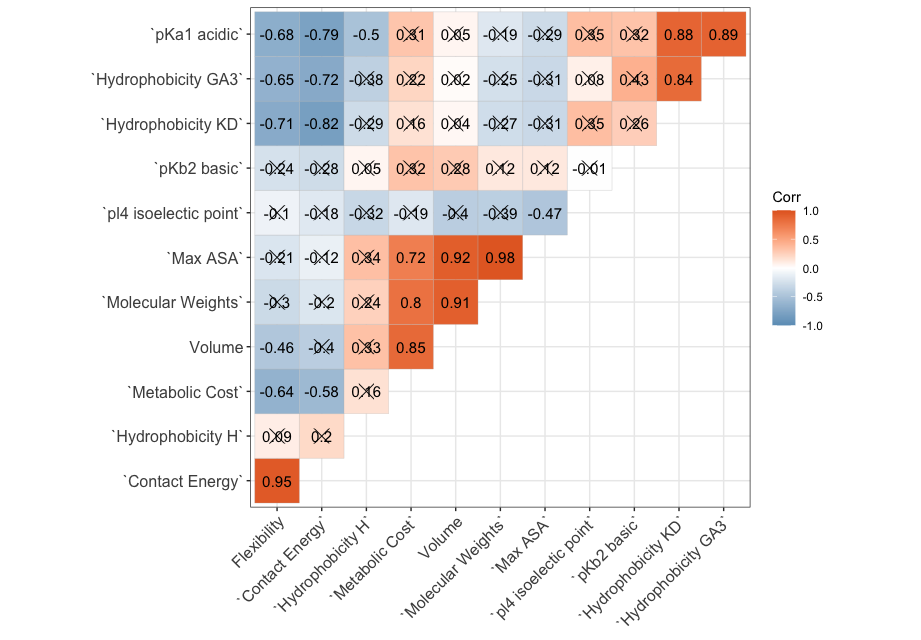
Supplementary Figure 1)

Correlation matrix between amino acid physicochemical properties. The strength of the correlation (in terms of Pearson’s R value) is indicated by colour saturation, red indicating a positive correlation and blue indicating a negative correlation; Pearson’s R values are also shown as numerical values in the matrix. Correlations that are not statistically significant have been marked though with an ‘X’.


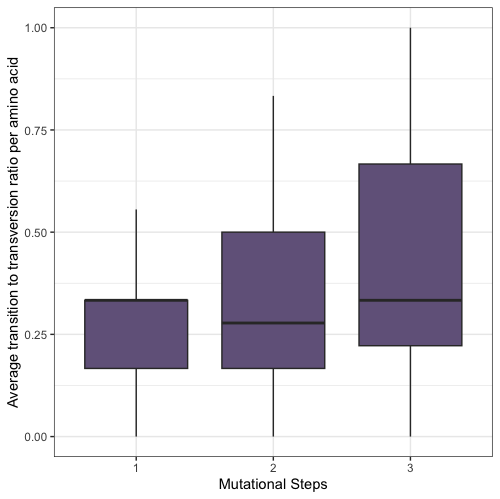


Supplementary Figure 2)

Boxplot showing the absolute difference in the average proportion of transition to transversion mutations separating pairs of amino acids, grouped by the number of mutational steps separating the amino acids. Boxes show the median, first and third quartiles of the data, while whiskers represent 1.5 * interquartile range.


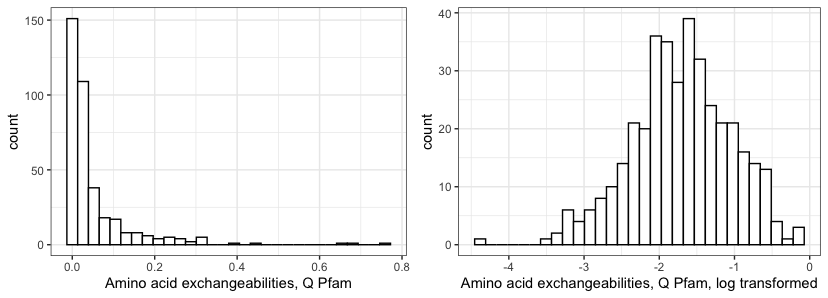


Supplementary Figure 3)

Distribution of amino acid substitution rates, as estimated from Pfam data, before and after log transformation.
